# Endocranial and masticatory muscle volumes in myostatin-deficient mice

**DOI:** 10.1098/rsos.140187

**Published:** 2014-12-17

**Authors:** Nathan Jeffery, Christopher Mendias

**Affiliations:** 1 Department of Musculoskeletal Biology, Institute of Ageing and Chronic Disease, University of Liverpool, Sherrington Building, Ashton Street, Liverpool L69 3GE, UK; 2 Department of Orthopaedic Surgery, University of Michigan, Ann Arbor, MI, USA

**Keywords:** masticatory muscles, brain, skull, evolution, mouse

## Abstract

Structural and functional trade-offs are integral to the evolution of the mammalian skull and its development. This paper examines the potential for enlargement of the masticatory musculature to limit the size of the endocranial cavity by studying a myostatin-deficient mouse model of hypermuscularity (MSTN−/−). The study tests the null prediction that the larger MSTN−/− mice have larger brains compared with wild-type (WT) mice in order to service the larger muscles. Eleven post-mortem MSTN−/− mice and 12 WT mice were imaged at high resolution using contrast enhanced micro-CT. Masticatory muscle volumes (temporalis, masseter, internal and external pterygoids) and endocranial volumes were measured on the basis of two-dimensional manual tracings and the Cavalieri principle. Volumes were compared using Kruskal–Wallis and Student's *t*-tests. Results showed that the masticatory muscles of the MSTN−/− mice were significantly larger than in the WT mice. Increases were in the region of 17–36% depending on the muscle. Muscles increased in proportion to each other, maintaining percentages in the region of 5, 10, 21 and 62% of total muscle volume for the external ptyergoid, internal pterygoid, temporalis and masseter, respectively. Kruskal–Wallis and *t*-tests demonstrated that the endocranial volume was significantly larger in the WT mice, approximately 16% larger on average than that seen in the MSTN−/− mice. This comparative reduction of MSTN−/− endocranial size could not be explained in terms of observer bias, ageing, sexual dimorphism or body size scaling. That the results showed a reduction of brain size associated with an increase of muscle size falsifies the null prediction and lends tentative support to the view that the musculature influences brain growth. It remains to be determined whether the observed effect is primarily physical, nutritional, metabolic or molecular in nature.

## 2. Introduction

The size and shape of most anatomical structures are rarely perfected during evolution and ontogeny for one specific purpose. More often, the final adult form represents a compromise between various competing demands superimposed on an underlying inherited bauplan. Mapping the distribution, nature and scale of these trade-offs is key to understanding, for example, the phylogenetic fidelity of traits commonly used in taxonomic reconstructions, phenotypic plasticity as well as the canalization of genetic diversity. Such interactions and trade-offs are particularly prominent in the head region—it must accommodate, among other things, the demands of the brain, the skull and the jaw muscles [1–3]. Evidence already exists linking, for example, changes in the orientation of the bony orbits, the cranial base angle and the angle between the petrous bones with relative differences of brain size [4–10]. Similarly, differences in the size and force production capabilities of the jaw-closing muscles have been linked to changes in the proportions of, for instance, the face, mandible and calvarium [11–15]. By contrast, there is comparatively little evidence to evaluate the proposition that the demands of the brain and masticatory muscles can pervade interceding adaptations of the skull to influence each other [16]. A trade-off between muscle and brain has been alluded to periodically in the literature for more than a century, typically formulated on the basis of broad trends seen across extant and extinct taxa and often explained in one of three ways: (i) as an artefact of allometry in which larger species tend to have proportionately smaller brains and larger masticatory muscles [17], (ii) that an enlarged brain, and the associated globular calvarium, cannot support enlarged musculature [18], and (iii) that enlarged muscles, and the forces they generate, constrain expansion of the brain [19,20]. The latter explanation implicit to Stedman *et al*.'s paper [16] has been the subject of considerable debate [21] but remains largely untested. Recently, Cray *et al*. [22] recognized that the myostatin-deficient mouse [23], with its hypermuscular phenotype, offered a potentially valuable model for testing the physical constraint hypothesis as it predicts a significant reduction of brain size with the increase of masticatory muscle size. Other mouse models have also proven useful in deciphering the relative importance of similar competing demands on the morphology of the mammalian skull [5,24,25]. The present study uses the myostatin-deficient mouse model to explore the possible interplay between the soft tissues of the brain within the skull and the masticatory muscles that envelope the skull.

The myostatin protein is a negative regulator of vertebrate muscle growth and disruption of the encoding gene results in large increases of skeletal muscle mass [23]. Myostatin signals through the type IB and IIB activin receptors and induces muscle atrophy by inhibiting the proliferation of muscle progenitor cells, and by activating proteolytic systems and inhibiting protein synthesis in mature muscle fibres [26–29]. Myostatin knock-out mice (MSTN−/−), therefore, have an overall increase in muscle and body mass through a combination of muscle fibre hypertrophy and hyperplasia [30]. Experiments in MSTN−/− mice have been used to infer the biomechanical influence of increased total muscle force on the skeleton [29,31–36]. Compared with wild-types, increases of absolute skeletal muscle force are in the region of 30–40% [30,37] and 30–60% increases of absolute bite force have also been recorded [31,38]. Documented changes of the skull linked to myostatin deficiency in mice include a compressed temporal bone, shorter cranial vault and longer cranial base [32,33,35]. Cray *et al.* [22] found no significant differences of cranial length, width, height and cranial volume in MSTN−/− mice on a CD1 background. However, the authors were limited by the radiographic methodology to estimates of cranial volume based primarily on ectocranial linear measurements. The investigation reported here extends their myostatin-deficient mouse model approach and evaluates the relationship with measurements of endocranial volume which is more intimately linked to the size of the brain. This study re-evaluates the hypothesis that enlargement of the masticatory muscles constrains enlargement of the underlying brain and its surrounding endocranial cavity by testing the null prediction that—*the larger myostatin-deficient mice* [22,35] *have the same sized, if not slightly larger, endocrania in comparison with wild-types in order to accommodate the increases of brain size needed to service the motor and proprioceptive requirements of a larger number of muscle fibres* [14,39].

## 3. Material and methods

### 3.1 Samples

All mice were of a C57BL/6J background. Wild-type (WT) mice were provided by Charles River Laboratories, USA. Myostatin-deficient mice (MSTN−/−) were originally provided by Dr Se-Jin Lee [23] and were backcrossed for several generations on a C57BL/6J background. Genotype of MSTN−/− mice was determined by polymerase chain reaction-based analysis of DNA obtained from tail biopsies at the time of weaning [30]. A total of 11 MSTN−/− adult male mice were sampled. To capture the range of adult variation, from sexual maturity through to osteological maturity, mice were taken at two (*n*=4), four (*n*=3) and 17 (*n*=4) months postpartum. Twelve WT mice were also sampled. To construct a broadly representative control sample, and hence provide a conservative test of MSTN−/− differences, the WT mouse sample consisted of four mixed sex mice (two males and two females) at age-matched intervals (2, 4 and 17 months). It is important to note that these sample sizes are not designed for reliable tests between age groups and genders but do capture a large range of murine variability and hence reduce the risk of falsely rejecting the null prediction.

### 3.2 Imaging and measurements

The head of each mouse was carefully removed and incubated for 10 days in formalin and 10% w/v I_2_KI prior to micro-CT imaging. This approach allows for the imaging of soft and hard tissues at high resolution (for details, see [40,41]). Specimens were imaged using the 320 kV open-bay X-Tek system at the University of Manchester (80–105 kV; 85–105 μA; 1440 projections). Data were reconstructed as isometric voxels with vertices between 32 and 37 μm long. Voxel data were imported into ImageJ (v. 1.46 [42]) and all volumes were measured independently by two observers using the VolumEst plugin (v. 20101201). This uses the Cavalieri principle to calculate volumes based on a subset of outlines manually drawn on orthogonal sets of two-dimensional slices (see also [43]). Volumes measured included the endocranial cavity and the jaw-closing muscles, namely the masseter, temporalis as well as the internal and external pterygoids. For convenience, the murine zygomaticomandibularis was considered part of the masseter. Outlines were taken every 70–350 μm depending on the size of the volume of interest. To test for a sampling bias, the first observer measured the endocranial volume every third coronal slice (approx. 105 μm) and the muscle volumes every second coronal slice (approx. 70 μm). The second observer measured the endocranial volume every tenth coronal slice (approx. 350 μm), the masseter and temporalis every fifth slice (approx. 175 μm) and the ptyergoids every third coronal slice (approx. 105 μm).

### 3.3 Statistics

Volumes from both observers were averaged and variations between MSTN−/− and WT volumes were then tested for using a standard Student *t*-test (assuming unequal variances if *F*-test indicated significant difference between variances). Data from both observers were then subjected to non-parametric Kruskal–Wallis tests to determine the significance of the observed differences between MSTN−/− and WT mice compared with the variance introduced by the observers and by differences of murine age and gender. All statistical tests were conducted in PAST (v. 3.01 [44]).

## 4. Results

**Figure 1.**
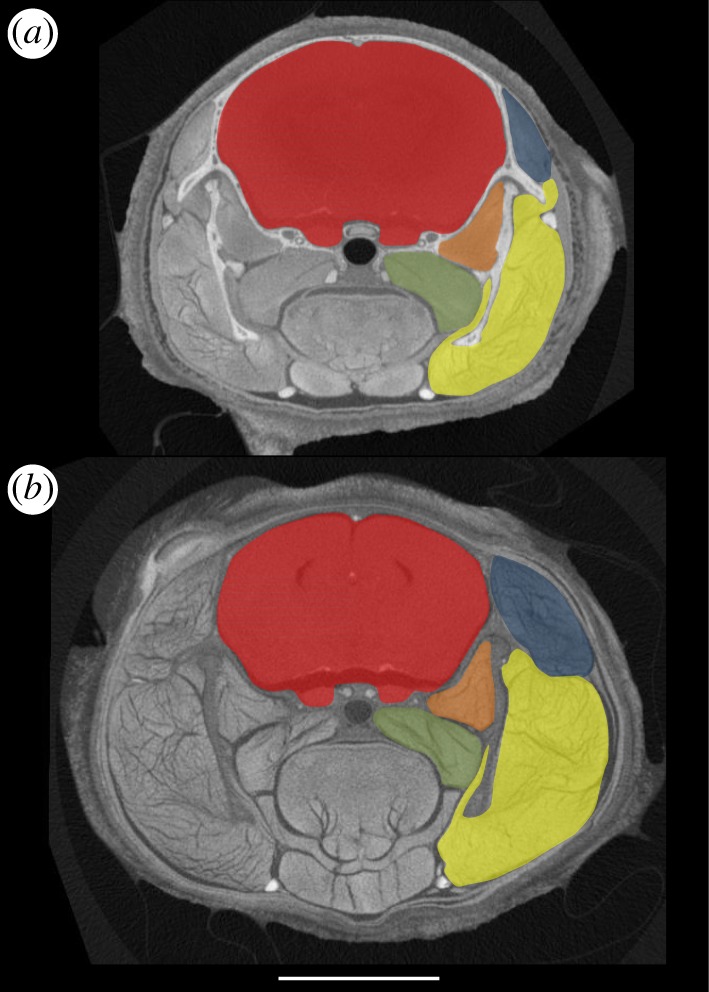
Coronal contrast-enhanced micro-CT slices through the head of four-month-old (*a*) WT and (*b*) MSTN−/− mice: red, endocranial cavity; blue, temporalis; yellow, masseter (including zygomaticomandibularis); green, internal pterygoid; orange, external pterygoid. Scale bar, 5 mm.

**Figure 2.**
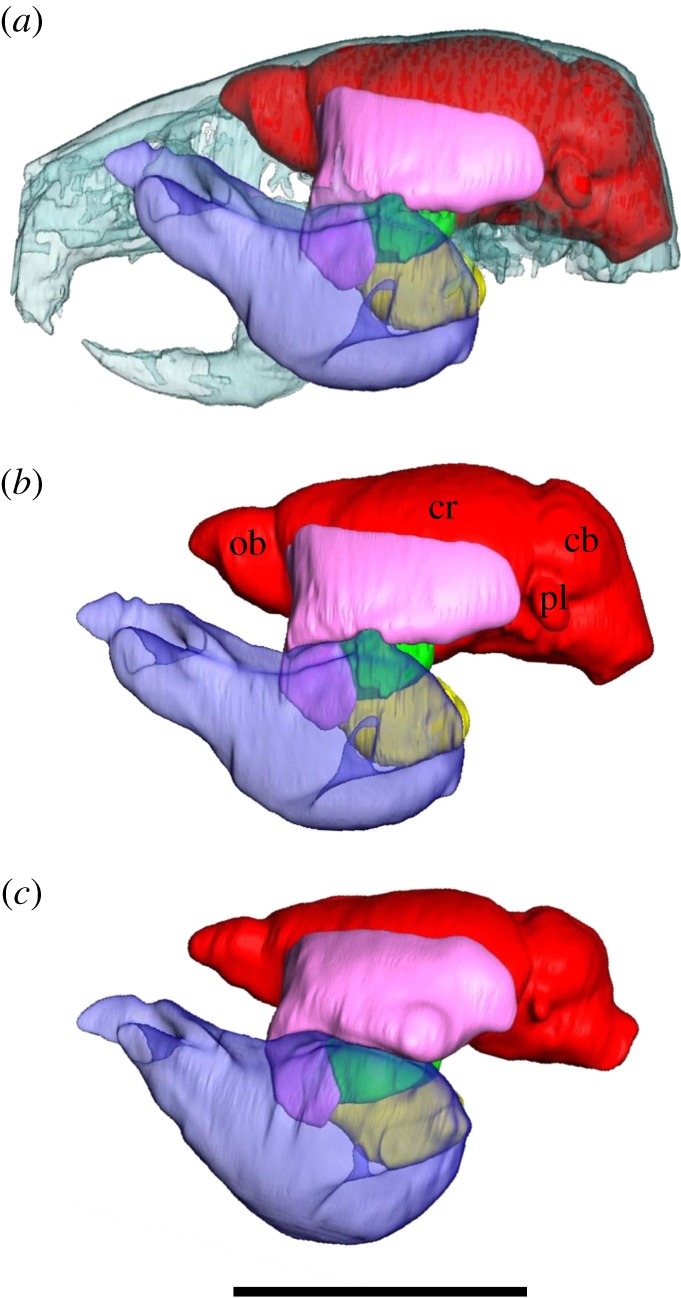
Lateral three-dimensional renderings of the masticatory muscles and endocranium in the (*a*) WT mouse (four months) superimposed on a semitransparent skull, (*b*) WT mouse (four months) and (*c*) MSTN−/− mouse (four months). Red, endocranium (ob, olfactory bulb; pl, petrosal lobule; cb, cerebellum; cr, cerebrum); pink, temporalis; transparent blue, masseter; yellow, internal pterygoid; green, external pterygoid. The distribution of thinner sections of muscle and their fascia across the skull is under-represented owing to volume-averaging during the three-dimensional rendering process. Scale bar, 10 mm.

Figure 1 shows reformatted two-dimensional slices through the head region and figure 2 shows three-dimensional reconstructions of the endocranium and masticatory muscles. These figures illustrate cross-sectional representations of the volumes of interest and give a snapshot of the differences between the two cohorts. Initial inspection of figure 2 suggests that the selected four-month-old MSTN−/− mouse (figure 2*c*) has a smaller endocranial volume as well as larger masticatory muscle volumes compared with the four-month-old WT mouse (figure 2*a*,*b*). Figure 2 also indicates that the reduction of size affects all regions of the brain, including for example, the petrosal lobules of the paraflocculus and the olfactory bulbs. Cohort average volumes and standard deviations are given in table 1.

**Table 1.** Endocranial and muscle volumes (mm^3^). *N*=24 WT and 22 MSTN−/− for means, s.d. and Kruskal–Wallis tests; *N*=12 WT and 11 MSTN−/− for Student's *t*-tests of pooled data.

	WT		% of total	MSTN−/−		% of total		Kruskal–Wallis
	mean	s.d.	muscle	mean	s.d.	muscle	*t*-tests	test
endocranial	503.3	26.1		434.8	20.3		^***^	^***^
external pterygoid	10.1	1.5	5	14.1	3.5	5	^**^	^**^
internal pterygoid	20.5	3.1	11	24.7	4.2	9	*	^**^
temporalis	41.2	10.0	21	64.7	10.8	23	^***^	^***^
masseter	119.9	24.9	63	172.0	35.4	62	^***^	^***^
total muscle	191.7	37.2	100	275.6	50.7	100	^***^	^***^

*p<0.05; ^**^p<0.01; ^***^p<0.001.

**Figure 3.**
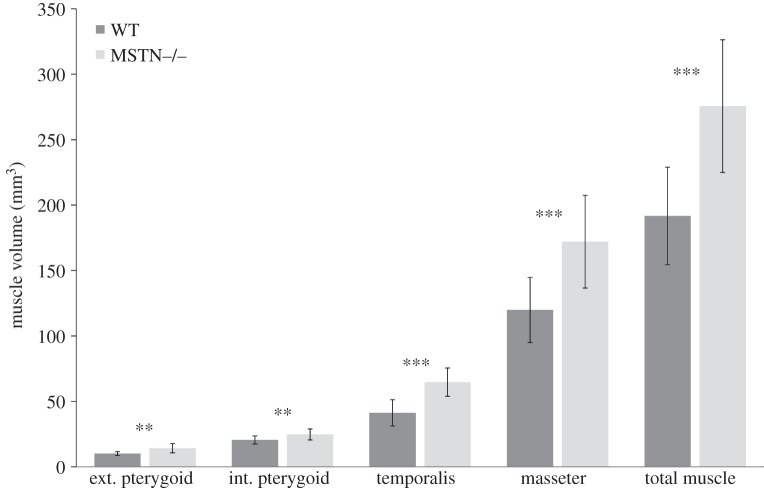
Bar graph plot of muscle volumes for MSTN−/− (light grey) and WT (dark grey) mice with +/− standard deviation error bars. Results of Kruskal–Wallis tests (table 1) are indicated (ns, not significant; *p<0.05; ^**^p<0.01; ^***^p< 0.001).

**Figure 4.**
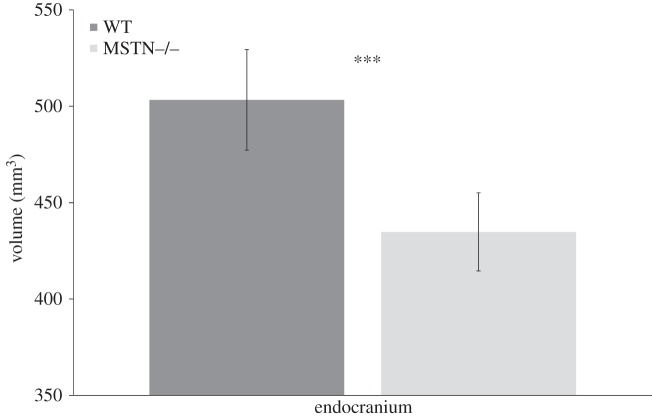
Bar graph plot of endocranial volumes for MSTN−/− (light grey) and WT (dark grey) mice with +/− standard deviation error bars. Result of the Kruskal–Wallis test (table 1) is indicated (ns, not significant; *p<0.05; ^**^p< 0.01; ^***^p<0.001).

**Figure 5.**
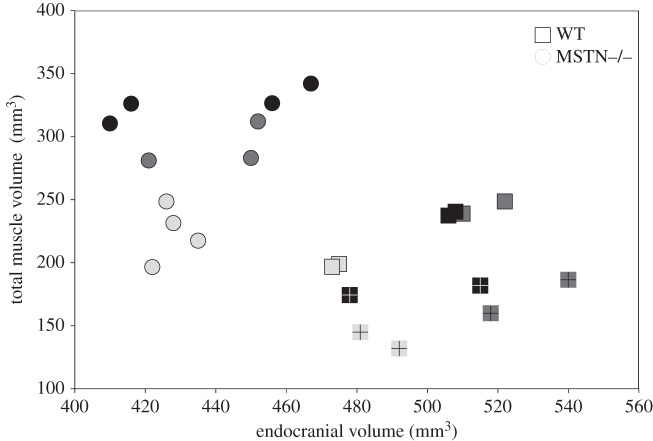
Bivariate plot of pooled endocranial and total muscle volumes for each mouse studied. Circles represent MSTN−/− mice; squares represent WT mice; light grey is 2-month-old, dark grey is 4-month-old and black is 17-month-old mice; females are marked with a cross symbol.

Figure 3 shows bar plots for averaged muscle volumes in WT and MSTN−/− mice and figure 4 shows a bar plot for endocranial volumes for each group. Table 1 gives the volumes and standard deviations. The *t*-tests (table 1) revealed significant differences between WT and MSTN−/− mice for endocranial volume and total muscle volume. These findings showed that on average MSTN−/− mice had significantly smaller endocranial volumes and significantly larger masticatory muscles compared with WT mice. Figure 5 shows a plot of observer averages of total muscle volume and of endocranial volume for each mouse studied. Datum points are coded for males and females as well as for the different age groups. At no point was there a significant overlap in the morphospace between the WT and the MSTN−/− mice. Results from the non-parametric Kruskal–Wallis tests confirmed that the differences between MSTN−/− and WT mice were significantly greater than differences attributable to observer variability and, for example, the age and gender of the mice.

## 5. Discussion

This study set out to investigate the ability of the murine skull to accommodate increases of masticatory muscle size while maintaining endocranial capacity. Both WT mice and MSTN−/− mice were examined. The MSTN−/− mice were characterized by significant increases of masticatory muscle size, allowing us to test for associated changes of endocranial capacity. The principal findings are discussed below.

Wild-type C57BL/6J muscle proportions (%) reported here were comparable to those reported by Baverstock *et al.* [45] for adult WT BALB/c mice. It is interesting to note that the percentage muscle proportions were similar for the MSTN−/− mice as well. This suggests that the knock-out had a uniform effect and that enlargement followed the underlying murine pattern. That there were significant volumetric differences between the WT and MTSN−/− masseter muscles is consistent with measurements of muscle mass [22,35]. The absolute WT muscle volumes differ compared with those reported by Baverstock *et al.* [45]. This probably reflects shrinkage due to the higher I_2_KI concentration and incubation period used in this study [46]. The inclusion of females in the present WT cohort and the staining method may have over- or underestimated the true extent of muscle size differences and readers should refer to the papers cited above.

Measurements of endocranial volume reported here were defined by the rigid, bony walls of the calvarium (figure 1) and were unlikely to have been affected by I_2_KI soft-tissue shrinkage. WT endocranial volumes were consistent with brain volumes previously reported for the same C57BL/6J strain and measured using *in vivo* magnetic resonance imaging [47,48], confirming that endocranial size is a reasonable proxy for brain size. Findings reported here demonstrated that the WT brain is approximately 16% larger than the equivalent MSTN−/− brain. The difference of endocranial size was evident in the micro-CT sections (figure 1), three-dimensional reconstructions (figure 2) and was statistically greater than variation due to age, gender and observer variability. The experimental design accommodated a broad range of brain size variation within the WT cohort in order to secure conservative tests of significant differences. As such the study design is arguably susceptible to false acceptance of the null predication but false rejection is less likely. Indeed, because the WT sample contained females, which on average have smaller brains than males [49] (though see also figure 5), this study may have underestimated the reduction of brain size in the exclusively male MSTN−/− cohort. The difference of brain size was almost an order of magnitude above that reported for sexual dimorphism (approx. 2.5%) and for different C57BL/6J populations [49,50]. That the MSTN−/− mice have smaller brains represents a significant new finding. Further investigations are needed to confirm whether, as indicated by figure 2, the observed size reduction is uniform or is concentrated in regions of the brain adjacent to structurally important parts of the skull (e.g. [51]).

Although it is tempting to attribute the reduction of brain size to the enlargement of the masticatory muscles, it is important to note that the evidence presented here does not conclusively prove a direct, structural, link as conceived by, for example, Stedman *et al.* [16]. There are several alternative explanations that warrant consideration—especially, if it were subsequently confirmed that regions of the brain such as the petrosal lobule are also reduced in size and are, as might be imagined for a structure embedded in the petrous temporal bone [52], less labile to the physical demands of the masticatory musculature. Simple body size scaling is one such alternative interpretation. Among mammals, brain size typically scales with negative allometry while the masticatory apparatus scales with positive allometry or isometry [53,54]. Hence, larger species tend to have larger brains in absolute terms but these appear smaller when expressed as a proportion of body size. For scaling to account for the absolute reduction of brain size reported here, the MSTN−/− mice would need to be significantly smaller than the WT cohort. Average MSTN−/− adult body mass is approximately 20% larger not smaller than equivalent WT mice [22,35]. Body size scaling seems an unlikely explanation for the absolute reduction of brain size reported here. It might also be imagined that there is an indirect trade-off in the energetic costs of building and maintaining these tissues (see premise in [39]) and that the additional burden of the enlarged musculature constrained the resources available to the developing brain. Typically, the brain takes nutritional priority under such circumstances (e.g. [55,56]) and maintains a similar proportion of total body mass. Interestingly, the rerouting of resources to support the nutritional demands of brain growth may partly explain the lower body fat composition reported for MSTN−/− mice compared with WT mice [57]. Anabolic agents formed by the MSTN−/− musculature might also influence the availability and/or structure of lipids within nervous tissue, leading to reductions of brain size. Cross-sectional area measurements of the lipid rich myelin sheath in the peripheral nervous system showed an average increase of 50% for MSTN−/− mice compared with WT mice [39]. Whether myelin area is also enlarged in the central nervous system remains to be determined, but if corroborated suggests that the brain should be larger not smaller in MSTN−/− mice. It is also conceivable that myostatin is more directly involved in brain growth. Studies have found the expression of myostatin in the mammalian hippocampus, cerebral cortex and the olfactory bulbs [58,59]. Although its exact function in these tissues remains unclear, Iwasaki *et al*. [59] have recently speculated that myostatin inhibits neurogenesis. Again, if correct this would imply that brain size should be comparatively larger in the knock-out and not smaller as reported here.

One final point to consider is the chronology of any proposed physical, nutritional or metabolic constraint. It is often reported that the mammalian brain achieves much of its adult size long before the musculature has had time to grow, limiting the window of opportunity for any putative constraint to occur during ontogeny [21,60]. The masseter muscle, for example, does indeed lag behind in control mice, reaching its adult size at around 50–60 postnatal days [22] compared with about 15–18 days for the brain [61,62]. Neonatal C57BL/6J brain volumes reported by Lee *et al*. [63] are 93 mm^3^, which is approximately 20% of the WT adult values reported here and elsewhere [47,48]. This implies that there is a period of accelerated brain growth (approx. 80% of the adult total) that coincides with the rapid divergence between control and MSTN−/− masseter weights seen from 0 to 28 days [22] as well as with the transition from placental sustenance to suckling and then to solid food at around 17–22 postnatal days [64]. It is conceivable that the murine brain is particularly sensitive to punctuated shifts of muscle size, activity and physiology during this accelerated phase of brain development rather than the relative timings of size maturation. Further studies of early postnatal and prenatal samples should help shed light on if and how the masticatory muscles might constrain brain growth.

## 6. Conclusion

The null prediction that myostatin mice have larger brains in order to service a larger number of muscle fibres was falsified. The study demonstrated a significant decrease of endocranial size in MSTN−/− mice compared with WT mice that exceeded age and gender differences. The present study did not, however, prove that this decrease is physically linked with the increase of muscle size characteristic of myostatin-deficient mice. Although we cannot conclusively resolve the aetiology of these findings, the discovery that the myostatin-deficient mouse brain is smaller represents a significant contribution to our knowledge of this phenotype and may have important repercussions for the development of therapies involving myostatin inhibition (e.g. [65]).

## Supplementary Material

Volume data for WT and MSTN-/- mice

## Data Accessibility

Muscle and endocranial volume data are available in the electronic supplementary material section.
