# Supplementary material for: Endocranial and masticatory muscle volumes in myostatin-deficient mice
Source: R Soc Open Sci. 2014 Dec 17;1(4):140187. doi: 10.1098/rsos.140187 (PMC4448778; doi:10.1098/rsos.140187)
Supplement: Volume data for WT and MSTN-/- mice [file rsos140187supp1.pdf]

				cubic mm					
**CODE**	**WT/MSTN-/-**	**Sex**	**Age (month**	**Endocranial Volume**	**Masseter Volume**	**Temporalis Volume**	**Internal Pterygoid Volume**	**External Pterygoid Volume**	**Total Muscle Volume**
2f1	WT	F	2	492	85	23	15	8	132
2f2	WT	F	2	481	93	28	15	8	145
2m1	WT	M	2	490	120	43	20	9	191
2m2	WT	M	2	482	119	43	19	9	189
4f1	WT	F	4	540	113	41	22	10	186
4f2	WT	F	4	518	97	35	19	10	160
4m1	WT	M	4	510	144	51	23	11	229
4m2	WT	M	4	522	148	52	24	10	235
17f1	WT	F	17	515	113	38	21	10	182
17f2	WT	F	17	478	109	35	20	10	174
17m1	WT	M	17	506	148	52	24	13	237
17m2	WT	M	17	508	150	54	24	13	240
MSTN4207	MSTN-/-	M	2	435	132	52	22	11	217
MSTN4206	MSTN-/-	M	2	422	121	47	19	10	197
MSTN4205	MSTN-/-	M	2	428	142	59	20	11	231
MSTN4204	MSTN-/-	M	2	426	155	59	22	12	249
MSTN4198	MSTN-/-	M	4	452	187	70	25	15	297
MSTN4195	MSTN-/-	M	4	421	168	60	23	13	264
MSTN4197	MSTN-/-	M	4	450	172	65	24	13	273
MSTN250	MSTN-/-	M	17	467	210	83	31	18	342
MSTN249	MSTN-/-	M	17	456	203	73	31	21	327
MSTN248	MSTN-/-	M	17	410	199	71	25	16	310
MSTN247	MSTN-/-	M	17	416	204	75	31	17	326
